# Aging deteriorates the ability to discriminate the weight of an object during an action observation task

**DOI:** 10.3389/fnagi.2023.1216304

**Published:** 2023-08-07

**Authors:** Andrea Albergoni, Monica Biggio, Emanuela Faelli, Piero Ruggeri, Laura Avanzino, Marco Bove, Ambra Bisio

**Affiliations:** ^1^Department of Neuroscience, Rehabilitation, Ophthalmology, Genetics and Maternal Child Health, Università Degli Studi di Genova, Genoa, Italy; ^2^Centro Polifunzionale di Scienze Motorie, Università Degli Studi di Genova, Genoa, Italy; ^3^Department of Experimental Medicine, Section of Human Physiology, Università Degli Studi di Genova, Genoa, Italy; ^4^IRCCS Policlinico San Martino, Genoa, Italy

**Keywords:** motor resonance, action observation, weight discrimination, aging, sensitivity, psychometric analysis

## Abstract

The ability to predict the weight of objects is important for skilled and dexterous manipulation during activities of daily living. The observation of other people moving objects might represent an important source of information on object features and help to plan the correct motor interaction with it. In aging, an impaired ability to evaluate the object weight might have negative drawbacks in term of the safety of the person. The aim of this study was to investigate the role of aging in the ability to discriminate the object weight during action observation. Twenty older adults (Old) and twenty young subjects (Young) performed a two-interval forced-choice task consisting in the observation of a couple of videos showing an actor moving a box of different weights. The observer had to evaluate which video showed the heavier box. Handgrip strength was acquired from all subjects. Sensitivity analysis was performed and psychometric curves were built on participants’ responses. The results showed a diminished sensitivity in the object weight discrimination in Old than in Young group. The analysis of the psychometric curves revealed that this impairment pertained both the light and heavy boxes and the minimum difference to discriminate different weights was greater in Old than in Young. At last, the sensitivity and the discrimination ability significantly correlated with individuals’ handgrip strength. These findings allow us to deeply characterize the impairments older adults have in discriminating the weight of an object moved by another individual.

## 1. Introduction

Instrumental activities of daily living (IADL) are complex actions required to maintain the independence in the social life (Wang et al., 2020). These activities include heavy household works as well as participating in cooperative task (Bowling et al., 2012; Edemekong et al., 2022). Examples of IADL that older adults frequently experience are moving and receiving objects, such as a box or a shopping bag. In the latter circumstance, it is important to recognize the characteristic of the object to adopt the correct motor strategy and being ready to receive it safely (Mizuguchi et al., 2016). In particular, the ability to predict accurately the weight of objects is essential for skilled and dexterous manipulation, and the observation of the features of the movement of other people lifting objects might represent an important source of information (Reichelt et al., 2013).

Action observation studies showed that both the weight of the object involved in the action and the effort of the observed individual influenced the activity of the observer’s sensorimotor brain areas (Meulenbroek et al., 2007; Alaerts et al., 2010a), suggesting that both characteristics are mapped into the individuals’ sensorimotor representation. This information is therefore used to plan the following motor response, as suggested by Reichelt et al. (2013), who showed that, after seeing the handling and the transfer of an object, the observer automatically adapted the lifting force to the weight of the observed object. An improper ability to evaluate the weight of an object might impair the individual during cooperative task and also could have negative drawbacks in term of the safety of the person.

Whether aging impacts on the ability to obtain information about the weight of an object lifted by an actor was examined in a previous study (Maguinness et al., 2013), which showed, in older adults, impairments when observing small and light (less than 1 kg) boxes, while no effect of aging was found for large and heavier (3÷18 kg) boxes. Since large boxes required full-body motion of the actor, authors claimed that these visual cues were more salient in term of information provided to the observer than that required to move the small boxes. However, the estimation ability was described only by the mean value of the sensitivity (*d’*) that, in case of large boxes, might have not been sufficiently sensitive. Furthermore, in that study, weight estimation ability when observing light and heavy boxes was done by showing stimuli differing not only in weight but also in the kind of movement performed by the actor, and in the boxes dimensions, features that might have had a confounding effect. For these reasons, a more detailed analysis, taking into account the different weights and presenting stimuli differing in the box’s weight, and neither in the action performed, nor in the box dimension, could be more informative. Since actions involving objects in this range of weight might cause the loss of the individual’s stability, it is particularly important to pursue the matter.

Furthermore, although aging is associated with deterioration in visual motion perception that may impair the ability to process relevant motion cues (Billino et al., 2008; Insch et al., 2012), other aspects directly related to movement features might impact on the ability to discriminate the object weight. The decrease in muscle mass and strength in elderly people is a phenomena largely proved causing muscle weakness (Rantanen et al., 2002; Cruz-Jentoft et al., 2010; García-Hermoso et al., 2018; Wang et al., 2020). Since a number of studies showed that, during action observation, motor resonance mechanisms, namely the activation of the observer’s sensorimotor system during action observation (Rizzolatti et al., 1999), occur and influence the way the individual perceives the observed action (Calvo-Merino et al., 2006, 2010; Bisio et al., 2010, 2014), one might hypothesize that the reduction of strength typical of physiological aging influences the evaluation of the weight of the object involved in the observed action.

The purpose of this study is to deepen the role of aging in the ability to discriminate the object weight. To unveil this issue, a weight discrimination video task was proposed to a group of healthy young adults and a group of older adults, who were required to observe a video showing an actor performing an IADL task (i.e., lifting a box on a shelf). Results were correlated with handgrip strength to explore the role that muscle strength has in this ability. Taking into account the results of Maguinness et al. (2013), we hypothesized that elderly people might show impairments when observing the lightest weight. Furthermore, based on the huge literature showing the strong effect that the sensorimotor repertoire has on action perception, we also hypothesized that the decline in strength in old individuals might be mirrored in the deterioration of the weight estimation ability.

## 2. Materials and methods

### 2.1. Participants

An *a priori* power analysis was conducted using G*Power version 3.1.9.7 (Faul et al., 2007) to determine the minimum sample required to test the study hypothesis. The effect size was set at 0.25 considered to be medium using Cohen’s criteria (Cohen, 1992). A *F*-test assessing the interaction between the group (*n* = 2) and the measurements (weights; *n* = 6) was applied with a significance criterion of α = 0.05 and power = 0.95. The minimum sample size needed was *N* = 40 for detecting differences in discrimination sensitivity between groups and among measurements.

Forty volunteers participated in the experiment. Depending on age, they were assigned to two groups, older adults (Old; *n* = 20; male/females: 7/13; mean age ± SE = 71 ± 2 years) and young participants (Young; *n* = 20; males/female: 9/11; mean age ± SE = 25 ± 1 years). To check if a significant difference exists in the proportion of males and females in the two groups, a Chi-squared test was performed and the result showed that these values did not significantly differ [χ^2^(1, *N* = 40) = 0.9, *p* = 0.34]. Older adults were recruited through informative flyers that were distributed at facilities where adapted physical activity programs were performed. As inclusion criteria, a medical certificate of good health was required from each elderly person. No neuropsychological and cognitive evaluations were performed, lacks that could represent limitations of the present study. Participants from both groups had normal or corrected-to-normal vision. Written informed consent was obtained from all participants before data collection. The study was approved by the ethical committee of the University of Genoa (Comitato Etico per la Ricerca di Ateneo, no 2021/42) and was conducted in accordance with the Declaration of Helsinki.

### 2.2. Experimental procedure

The experiment consisted in a single session and included a strength measurement and weight discrimination video task. The strength (kg) of the participants was assessed with the KERN MAP handgrip dynamometer (KERN & SOHN GmbH). The participants performed the test in the standard position, sit on a chair, with their shoulder adducted and neutrally rotated and the elbow flexed at 90° (Innes, 1999). The mean value obtained from three repetitions allowed the assessment of the right and left handgrip strength (Innes, 1999). The mean values between left and right handgrip strength was considered as the outcome parameter of Handgrip Strength (HS).

**FIGURE 1:**
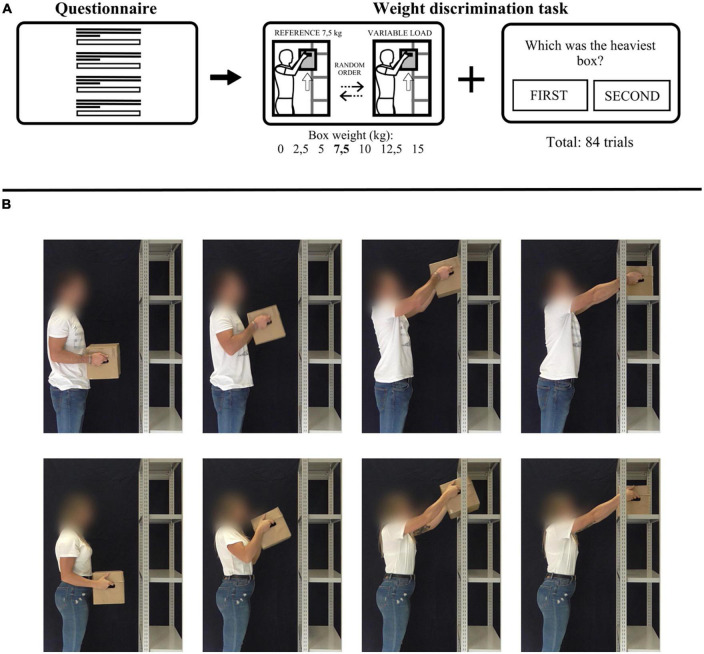
Experimental design. **(A)** Task protocol: each participant executed the task on the computer. The participants filled a questionnaire collecting personal and physical activity related data. Then a two-interval forced-choice (2IFC) task consisted of 84 trials. Each trial contained two videos in sequence: one was the reference stimulus (box weight: 7.5 kg) and the other was one of the seven comparison stimuli (box weights: 0, 2.5, 5, 7.5, 10, 12.5, 15 kg). At the end a question asked in which video the box was heavier, and the participants could choose between “first” or “second.” **(B)** Frames of one video showing the actor (top panel) and the actress (bottom panel) proposed to participants.

The weight-discrimination video task was built using jsPsych 6.3.0 library and performed off-line (de Leeuw, 2015). The task was preceded by the instructions, and by a questionnaire collecting personal data (i.e., sex, age, weight, height) and physical activity level (i.e., activities performed, year of experience, weekly hours of training) data. The protocol is represented in Figure 1A.

#### 2.2.1. Video stimuli

The stimuli consisted in videos showing an actress (26-years old, 1.67 m tall, 60 kg in weight) or an actor (28-years old, 1.72 m tall, 70 kg in weight) based on participant’s sex performing an IADL; in particular, they moved a box from the chest to a shelf over the head. The box was filled with varying amounts of sheets of paper in such a way as to assume seven different weights (0, 2.5, 5, 7.5, 10, 12.5, 15 kg). Actors were informed about the weight of the box. Videos, whose durations range from 1.3 to 3.7 s, were acquired in the same day with a video-camera positioned to record the execution of the lifting movement laterally. Both actors wore a white T-shirt with short sleeves and long jeans. The face of the actors was blurred to cover facial expression. Example frames of the video showed to participants are provided in Figure 1B.

#### 2.2.2. Task

Participants seated in front of a laptop with a 16-inch LCD screen position on a table, at a distance of about 60 cm. They were required to perform a two-interval forced-choice (2IFC) task (Duarte et al., 2018). Each trial consisted in a sequence of two videos: a reference and a comparison stimulus. After observing both videos, participants had to indicate in which video the box was heavier. In particular, subjects had to press the left arrow key to answer “First” and the right arrow key to answer “Second.” The 7.5 kg-video was showed every trial and it represented the reference stimulus. The 0-, 2. 5-, 5-, 7. 5-, 10-, 12. 5-, and 15- kg-video were the comparisons stimuli (please notice that 7.5 kg-video was used both as reference but also as comparison). In each trial, the order of appearance of the reference and the comparison stimuli was randomized. Each comparison video was displayed 12 times in a randomized order, for a total number of trials corresponding to 84 (7 box weights, 12 repetitions). The total duration of the task was about 25 min.

### 2.3. Data analysis

The discrimination sensitivity (*d’*) and the response bias (*c*) were evaluated at each comparison stimulus (except 7.5 kg) using signal-detection theory (Macmillan and Creelman, 2004). The *d’* was calculated according for one-dimensional classification experiments, following the procedure adopted by Norman et al. (2009). A “hit” occurred was when the first weight was heavier and the participants correctly responded “first was heavier.” A “false alarm” occurred when the first weight was lighter, but the participants incorrectly responded “first was heavier.” The hit rate was computed by dividing the number of hits by the total number of trials in which the first weight was heavier; the false-alarm rate was obtained by dividing the number of false alarms by the total number of trials in which the first weight was lighter (Norman et al., 2009). The log-linear adjustment method was used to adjust for extreme values of hits and false alarms (Macmillan and Creelman, 2004). The higher *d’* values, the better the ability to discriminate between the object’s weight. The measure of response bias (*c*) was used to determine whether participants showed a preference to use either the higher or the lower end of the weight scale. *Mean d’* and *mean c* were calculated by averaging the values at the different weights (except 7.5 kg).

The percentage of response in which the comparison stimulus was judge “Heavier” than the reference stimulus (hereafter Heavier probability) at each comparison stimulus (including 7.5 kg) was computed for each participant. These proportions were used to build a psychometric function. The observers’ psychometric curves were obtained by finding the best-fitting logistic functions using *psyphy* and *quickpsy* R package (Yssaad-Fesselier and Knoblauch, 2006; Linares and López-Moliner, 2016). The lower and upper asymptotes, threshold, and just noticeable difference (JND) were estimated for each psychometric function (Knoblauch and Maloney, 2012). Lower asymptote (A_LOW_) and upper asymptote (A_UP_) were computed according to Oh et al. (2016). The lower/higher A_LOW_/A_UP_, the better the ability to discriminate low/high weights. The threshold corresponds to the curve point crosses 0.5 on the *y*-axis and indicates the point of subject equality (Kopec and Brody, 2010). JND is considered as the smallest weight that produces changing in perception and calculated as the half difference between the weights at which the psychometric function equals to 0.75 and 0.25, respectively (von Sobbe et al., 2021). A lower JND indicated a better ability to discriminate the stimuli.

### 2.4. Statistical analysis

The Handgrip Strength (HS), sensitivity (*d’*) at each comparison stimulus (Norman et al., 2009), mean sensitivity (*mean d’*) (Maguinness et al., 2013), response bias (*c*) at each comparison stimulus, mean response bias (*mean c*) (Maguinness et al., 2013), Heavier probability at each comparison stimulus, A_LOW_, A_UP_, threshold, and JND were considered as outcome parameters. Shapiro-Wilk test was applied to evaluate data distribution and Levene’s test was used to evaluate the equality of variances. HS, *mean d’*, and *mean c* were normally distributed, whilst *d’*, *c*, Heavier probability, A_LOW_, A_UP_, threshold, and JND were not.

Left and right HS were statistically evaluated by means of a repeated measure ANOVA with GROUP as between subject factor (2 levels: Old and Young), and SIDE as within subject factor (2 levels: Right and Left). Then, since the task displayed in the video was a bimanual task, left and right HS were averaged in the following analyses.

Concerning the sensitivity analysis, Mann–Whitney tests were applied to compare *d’* and *c* values of both groups at each comparison stimulus between groups. Then, a *t*-test was performed to statistically compare *mean d’* and *mean c* between Old and Young. To account for the effects of age, partial correlation was applied to test the relationship between HS and *mean d’* on data from both groups pooled together.

Concerning the psychometric function, Heavier probability at each comparison stimulus (including 7.5 kg) was compared between groups by means of Mann–Whitney test. The not-normally distributed data derived from the curve were statistically evaluated by means of Mann–Whitney test with the aim to compare the two groups, and Wilcoxon test to assess differences between conditions within each group. To account for the effects of age, partial correlations were applied to assess the relationship between HS and threshold, JND, A_LOW_, A_UP_ on data from both groups pooled together.

Normally distributed data are reported as mean value ± standard error (SE), while not-normally distributed data are given as median [interquartile range (IQR)]. Significance level was set at 0.05. Statistical analyses were performed with IBM SPSS Statistics 26 software.

## 3. Results

### 3.1. Handgrip strength

The ANOVA applied to compare HS between two groups revealed a significant main effect of GROUP [*F*_(1,38)_ = 4.18, *p* = 0.048, η^2^ = 0.096], indicating that HS in Young (32.3 ± 1.0 kg) was significantly higher than in Old (23.3 ± 1.2 kg). A significant main effect of SIDE was also found [*F*_(1,38)_ = 14.42, *p* = 0.00005, η^2^ = 0.01], and Right HS (30.1 ± 1.2 kg) was significantly higher than Left HS (28.1 ± 1.1 kg).

### 3.2. Sensitivity analysis (*d’*)

**FIGURE 2:**
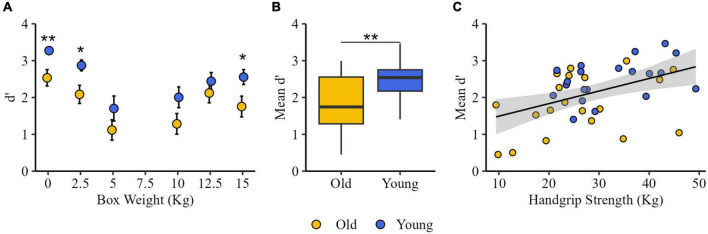
Sensitivity analysis. **(A)** Discrimination sensitivity (*d’*) for older adults (Old, yellow) and young participants (Young, blue) groups at each comparison stimulus (except 7.5 kg). **(B)** *Mean d’* values of both groups. The box represents the inter-quartile ranges, and the bars show the maximum and the minimum. **(C)** Correlations between handgrip strength and *d’*. Each dot represents the *d’* value as function of handgrip strength for each participant for Old and Young group. *p < 0.05, **p < 0.01.

Mann–Whitney tests showed that *d’* values were significantly higher in Young than in Old group at 0 kg (Old: 2.70 [2.05, 3.46]; Young: 3.46 [3.46, 3.46]; *Z* = −2.66, *p* = 0.008), 2.5 kg (Old: 2.16 [0.97, 2.89]; Young: 2.70 [2.70, 3.46]; *Z* = −2.24, *p* = 0.025), and at 15 kg (Old: 1.56 [1.37, 2.70]; Young: 2.70 [2.56, 2.89]; *Z* = −2.21, *p* = 0.027) (Figure 2A).

The statistical analysis on *mean d’* showed a significant difference between groups [t(38) = 3.07, *p* = 0.004] indicating that *d’* was higher in Young group (2.12 ± 0.10) than in Old group (1.56 ± 0.15) (Figure 2B). A significant positive correlation was found between mean HS and *mean d’* in (*R* = 0.35, *p* = 0.03) (Figure 2C).

### 3.3. Bias analysis (*c*)

The statistical analysis on response bias failed to find significant differences between the two groups both when considering *c* at each weight and when analyzing *mean c*.

### 3.4. Weight discrimination ability

**FIGURE 3:**
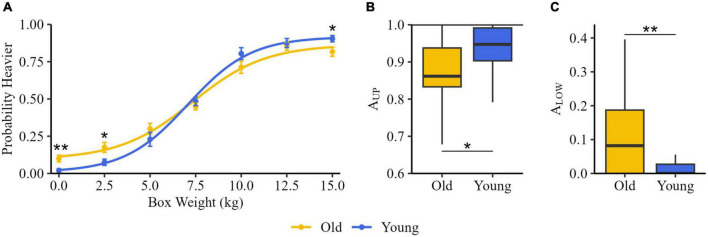
Weight discrimination. **(A)** Psychometric functions for older adults (Old, yellow) and young subjects (Young, blue). Dots represent the proportion of Heavier responses at comparison stimulus for each group, obtained from the average of the responses of all participants. **(B)** Lower asymptotes (A_LOW_) and **(C)** upper asymptotes (A_UP_) and for old and young groups. The black horizontal line indicates the median, the box represents the inter-quartile ranges, and the bars show the maximum and the minimum. *p < 0.05, **p < 0.01.

The graphical representation of the psychometric functions of the two groups is displayed in Figure 3A.

The comparison between Heavier probability at each box weight showed a significant main effect of GROUP at 0 kg (Old: 0.08 [0.00, 0.17]; Young: 0.00 [0.00, 0.00]; *z* = −2.94, *p* = 0.003), at 2.5 kg (Old: 0.17 [0.06, 0.27]; Young: 0.08 [0.00, 0.08]; *z* = −2.24, *p* = 0.025), and at 15 kg (Old: 0.83 [0.75, 0.92]; Young: 0.92 [0.90, 0.94]; *z* = −2.35, *p* = 0.019).

The result of the statistical analysis on A_LOW_ showed that it was significantly higher in Old (0.08 [0.00, 0.19]) than in Young (0.00 [0.00, 0.03]) (*U* = 115, *z* = −2.49, *p* = 0.021) (Figure 3B). Young’s A_UP_ was significantly higher than that of Old (*U* = 120, *z* = −2.36, *p* = 0.02; Old 0.86 [0.83, 0.94]; Young 0.95 [0.90, 0.99]) (Figure 3C).

Concerning the threshold, no difference between groups was found (Old: 8.07 [6.59, 9.39] kg; Young: 7.51 [7.34, 7.66] kg).

**FIGURE 4:**
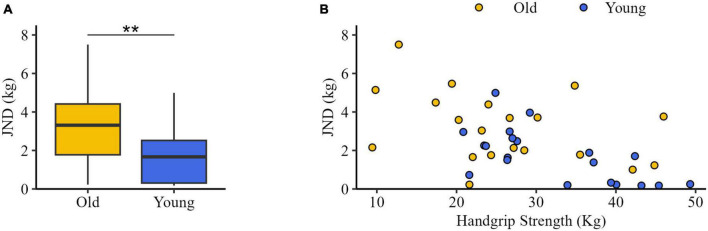
Just noticeable difference (JND). **(A)** JND values for older adults (Old, yellow) and young subjects (Young, blue). The black horizontal line indicates the median, the box represents the inter-quartile ranges, and the bars show the maximum and minimum value. **(B)** Correlations between handgrip strength and JND. Each dot represents the JND value as function of handgrip strength for each participant of both groups. **p < 0.01.

JND value was significantly higher in Old (3.31 [1.77, 4.41] kg) than in Young (1.67 [0.31, 2.52] kg) (*U* = 107, *z* = −2.53, *p* = 0.010) (Figure 4A). A significant negative correlation was found between mean HS and JND (*R* = −0.40, *p* = 0.011) (Figure 4B).

## 4. Discussion

The results of this study showed that human ability to discriminate the weight of a box moved by an actor significantly deteriorates due to aging for different amount of weight. In particular, after confirming the literature showing a general diminished sensitivity for discriminating the object weight with respect to young subjects (analysis on *mean d’*) (Maguinness et al., 2013), the analysis on *d’* and on the psychometric curves allowed us a more in-depth characterization of this phenomenon with respect to previous studies, which took into account differences between groups for different amount of weight. In particular, the analysis on *d’* at the different weights showed that the sensitivity of Old group was significantly lower than that of Young group for the both the lighter (0 and 2.5 kg) and the heaviest (15 kg) boxes. In agreement with this result, the point-by-point analysis of the psychometric functions showed that older adults’ discrimination ability was lower than that of young participants in correspondence of the same weights. Impairments in weight discrimination for both light and heavy boxes are confirmed by the analysis of the asymptotes of the curve, which give information about the ability to discriminate extremely low and high weights. Indeed, the values of the lower asymptote (providing information about the ability to judge light boxes) were higher for Old than for Young, whilst the value of the upper asymptote (providing information about the ability to judge heavy boxes) was lower for Old than for Young. Furthermore, JND values were significantly higher in Old than in Young, suggesting that the minimum difference to discriminate different weights was greater in the elderly population than in young subjects. At last, the correlation analyses showed that the discrimination ability evaluated by means of *mean d’* and JND significantly correlated with individuals’ handgrip strength regardless of age.

The results described by the sensitivity analysis on *mean d’* are in line with those of the literature exploring the effects of aging on weight discrimination, and showed that older adults’ sensitivity for discriminating the different weights is impaired with respect to that of young subjects (Watson et al., 1979; Norman et al., 2009; Maguinness et al., 2013). This finds confirmation in the JND parameter resulting from the psychometric curves, whose value in Old was about twice as much as those of Young, indicating that, for this kind of movement, the minimum difference to discriminate the weight of two moving objects is higher in the elderly population. No differences were found in *mean c* and *c*, suggesting no response bias in weight estimation in the two groups.

The results of sensitivity analysis at each comparison stimulus and of the psychometric analysis add new insight on this matter. Indeed, for the first time, this study provides an in-depth quantification of elderly’s ability to evaluate different amount of weight during action observation. For both light and heavy boxes, *d’* was worse in older adults than in young participants. This was confirmed by the point-by-point analysis of the psychometric functions and the analysis on the curve asymptotes, which revealed that differences in the accuracy of the response were found for both light and heavy boxes. This result is at odds with those described in Maguinness et al. (2013) study. In that work, authors failed to find differences in *mean d’* between young and old subjects when showing the lifting of a large box, the weight of which ranged from 3 to 18 kg. The box displayed in the present study was quite similar in dimensions to that used in the “large box condition” of Maguinness et al. (2013) work and the highest weight, namely 15 kg, is in the range of weight they used. However, authors did not test *d’* at the different weights within the range. Therefore, one cannot exclude that their results would have been different if a point-by-point analysis had been performed.

Having a detailed description of what happened for different weights is particularly relevant to be considered in case of heavy objects. Indeed, during cooperative tasks it is common to receive objects from a companion. To do it efficiently and safely, our brain extrapolates the information from the companion’s motion and use it to scale forces to cope with the expected load (Reichelt et al., 2013). If the mechanisms involved in weight estimation is impaired, the individual could adopt an inappropriate motor strategy, making the interaction less effective and putting the own neuromuscular system integrity at risk.

It is known that movement perception triggers motor resonance mechanisms, namely the activation of the perceiver’s sensorimotor system when observing someone else actions (Rizzolatti et al., 1999). In turn, this mechanism is influenced by the own sensorimotor experience that models the way the individual perceives the external word (Aglioti et al., 2008; Bisio et al., 2010; Petroni et al., 2010). The possibility for the observer to create a direct match (Rizzolatti et al., 2001) between the own sensorimotor repertoire and the observed action might thus be influenced by the different sensorimotor capacities of the actor and the observer. Specifically concerning the role of motor resonance in estimating the weight of an object moved by an actor, in a recent study it was shown that sensorimotor expertise in weight lifting improves the perceptual weight estimation ability (Albergoni et al., 2023). On the other hand, an alteration of the sensorimotor repertoire, as may occur during aging, might negatively affect the perception of the kinematic features of the observed movement that underlies object weight estimation (Alaerts et al., 2010b,a). In the present study, older adults had a significantly lower handgrip strength with respect to young subjects. For the first time, this parameter was shown to influence participants’ ability to discriminate the weight of the moved object. In particular, the positive correlation that appeared between handgrip strength and *mean d’*, suggests that individuals who developed higher force were those with a higher sensitivity in discriminating the observed object weight. In agreement, the negative correlation between HS and JND indicates that the least noticeable difference between two weights decreased with increasing strength. Therefore, the higher the strength of the individual, the better the ability to discriminate the object weight during an action observation task, even when controlling for age. Thus, one might conclude that the deterioration of the strength capacity in aging affects the individual’s ability to perceive the weight of an object moved by another person.

To explain the differences between groups one cannot exclude the contribution of other factors. Healthy aging is characterized by a decline in the neural system that pertains both central and peripheral regions of the nervous system, causing impairments in motor control and in movement perception (Seidler et al., 2010; Hunter et al., 2016). In particular, at peripheral level, the proprioceptive receptors, in charge of sending to the brain information related to the sense of position, strength and heaviness (Proske and Gandevia, 2012), undergo anatomical and physiological changes during aging that cause a deterioration of the proprioceptive afferences (Shaffer and Harrison, 2007; Goble et al., 2009). This altered input reaches the sensorimotor brain regions that, in turn, suffer from aging, resulting in proprioceptive dysfunctions (Goble et al., 2009). All these physiological changes, known to contribute to a decline in motor control (Seidler et al., 2010), might have negatively affected also motion perception (Roudaia et al., 2010). Furthermore, deterioration in the processing of biological motion displays (Billino et al., 2008; Pilz et al., 2010; Insch et al., 2012) and in the sensitivity to changes in the speed of a moving stimuli present with aging (Conlon and Herkes, 2008) have been called into question. Since no comparisons were made with other perceptual tasks in this study, it remains unknown whether the decline associated with aging is specific to the current task or if it could be generalized to other perceptual tasks. This is a limitation of the present study. At last, the brain regions involved in weight perception in both frontal and parietal lobules (Hamilton et al., 2006) are known to undergo to progressive deterioration in aging that can explain these findings (Seidler et al., 2010).

In conclusion, the present findings provide an in-depth characterization of the effects of aging on the ability to discriminate the weight of an object moved by another person, by showing, for the first time, an impairment for both light and heavy objects and that this ability depends on the force the individual is able to express. Since the ability to estimate the weight of a moved object can influence the individual’s daily life activity, one might suggest that working on strength training can be beneficial not only to improve movement execution but also in perceptual task, such as object weight estimation.

## Data availability statement

The raw data supporting the conclusions of this article will be made available by the authors, without undue reservation.

## Ethics statement

The studies involving human participants were reviewed and approved by the Comitato Etico per la Ricerca di Ateneo, (no 2021/42), University of Genoa. The patients/participants provided their written informed consent to participate in this study.

## Author contributions

AA, MBo, and AB involved in the conceptualization. MBi and EF contributed to the methodology. AA performed the experiments. AA, MBi, LA, MBo, and AB involved in the data analysis. MBi, EF, PR, and MBo provided interpretive analysis and critical revisions. AB supervised the original research. AA and AB wrote the manuscript. All authors contributed to the revisions of the manuscript and approved the final manuscript.
